# Semi-Synthesis and Biological Evaluation of Novel Sinomenine Derivatives

**DOI:** 10.3390/molecules30183802

**Published:** 2025-09-18

**Authors:** Meichun Wu, Zhewei Zhang, Ze Li, Zijian Zhao

**Affiliations:** 1 College of Chemistry and Materials Engineering, Huaihua University, Huaihua 418000, China; cq0078@163.com (M.W.); zzw010718@163.com (Z.Z.); a187z3423788@163.com (Z.L.); 2 Hunan Provincial Engineering Technology Research Center for Polyvinyl Alcohol Based New Functional Materials, Huaihua 418000, China; 3 Key Laboratory of Research and Utilization of Ethnomedicinal Plant Resources of Hunan Province, Huaihua University, Huaihua 418000, China

**Keywords:** sinomenine derivatives, molecular docking, druggability analysis, analgesic activity

## Abstract

Sinomenine has long been known as an anti-inflammatory drug, while poor efficiency and large-dose treatment had limited its further application. Five novel sinomenine 1-Br-4-cinnamic acid esters derivatives **2a**–**2e** were designed and synthesized to improve its analgesic and anti-inflammatory activity. All synthesized sinomenine derivatives were structurally confirmed by NMR and ESI-MS. Molecular docking results showed that compounds **2a**–**2e** had stable binding to the GBP5 protein. The compounds **2a**–**2e** showed stable binding to the GBP5 protein by molecular docking Pre-preparing the druggability of compounds **2a**–**2e** by ADEMT 3.0 showed that each derivative had similar druggability to sinomenine. The analgesic activity of compounds **2a**–**2e** was preliminarily determined by hot plate and acetic acid writhing experiments, while anti-neuroinflammatory effects were evaluated by a xylene-induced mouse ear edema model. The results of the hot plate method showed that the synthesized sinomenine derivatives **2a**–**2e** had some analgesic effects. The results of the acetic acid writhing test showed that the analgesic effects of **2a**, **2c**, **2e** were better than that of sinomenine, and the other derivatives were equivalent to sinomenine. Compound **2b** showed excellent anti-inflammatory properties in mouse ear edema.

## 1. Introduction

Sinomenine (SIN) is a bioactive isoquinoline alkaloid extracted from the medicinal plant Sinomenium acutum [1], which has a wide range of pharmacodynamic properties, including anti-inflammatory, antioxidative, antisynovitis, antineoplastic, immunosuppressive, antihypertensive, and antiarrhythmic effects, and exerts therapeutic effects on rheumatoid arthritis in clinical application [2,3,4,5]. Therefore, SIN was regarded as a lead compound in drug research, especially for anti-inflammatory medicines. However, some side effects, for example, hydrophilic–lipophilic balance (HLB), large dose allergies, and the induction of hypersusceptibility [6,7,8,9,10], prevent it from becoming a drug directly. In order to obtain sinomenine analogs with better therapeutic effects and fewer side effects, structural modifications of sinomenine have attracted significant research to improve its further applications [11,12,13].

Structurally, SIN is an isoquinoline alkaloid with a tetracyclic structure derived from the morphine backbone, featuring fused rings that include an isoquinoline core and structural characteristics typical of opioid alkaloids. It contains a number of functional groups, methoxyl, such as aryl, double bond, carbonyl, phenolic hydroxy, and N-methyl. Thus, it is very suitable for modification research [6]. Furthermore, SIN possesses a well-designed substitution pattern on ring A. The free sinomenine phenolic hydroxyl on ring A is readily oxidized by air and may be the main cause of hypersusceptibility [14]. To improve activity, the structural modification of sinomenine has attracted enormous interest in recent years [15,16].

The typical symptoms that can be induced by inflammatory stimuli mainly include fever, pain, redness, and swelling [17]. Despite the existence of studies on the relieving effect of sinomenine derivatives, the potential anti-inflammation effects of sinomenine derivatives in xylene-induced ear edema still remain poorly investigated and understood [15]. Xylene can promote the generation and transmittance of TNF-α, IL-1β, and inflammatory mediator PGE2, leading to acute exudative inflammatory edema in the mouse ear, with a significant increase in the rate of change in ear weight and ear thickness. To establish a xylene-induced mouse ear edema, an in vivo inflammatory model is beneficial to a more comprehensive study of the inhibitory mechanism of sinomenine derivatives on inflammation.

In this article, a series of sinomenine 1-Br-4-hydroxy esters with cinnamic acids are designed based on the prodrug principle and the synergistic effect for some cinnamic acids and their derivatives possessing good analgesic and anti-inflammatory activity [18]. Furthermore, the analgesic effects of the sinomenine derivatives were investigated by the hot plate method and the acetic acid-induced writhing test, while a mouse ear edema model induced by xylene was established to investigate the anti-inflammatory impact of various sinomenine 1-Br-4-cinnamic acid ester derivatives at certain dosages. These esters were expected to possess more efficient analgesic and anti-inflammatory activity effects with better bioavailability and HLB [19].

## 2. Results and Discussion

### 2.1. Chemical Synthesis

**Scheme 1:**
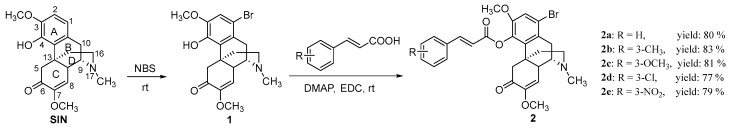
Synthesis of compounds **2**.

Compounds **2a**–**2e** were prepared following the procedure presented in Scheme 1. SIN reacted with N-bromosuccinimide (NBS) in dry DCM to yield compound **1**. As the H-1 of SIN was easily halogenated by NBS, the SIN-1-Br was prepared. Then, it reacted with different substituted cinnamic acids in the presence of EDC and DMAP to yield compounds **2**. NMR analysis confirmed the successful synthesis of compounds **2a**–**2e**.

1-Br-4-cinnamic acid ester-sinomenine **2a:** white solid, 80% yield; m.p. 35–38 °C; ^1^H NMR (400 MHz, CDCl_3_) *δ* 8.02 (d, *J* = 16.3 Hz, 1H), 7.65 (d, *J* = 3.4 Hz, 1H), 7.58 (d, *J* = 8.2 Hz, 1H), 7.45–7.39 (m, 2H), 7.07 (s, 1H), 6.68 (d, *J* = 16.1 Hz, 1H), 5.46 (s, 1H), 3.92 (d, *J* = 15.1 Hz, 1H), 3.72 (s, 3H), 3.50 (s, 3H), 3.24 (t, *J* = 4.6 Hz, 1H), 3.06 (d, *J* = 18.8 Hz, 1H), 2.99 (s, 1H), 2.50 (dd, *J* = 17.8, 6.1 Hz, 3H), 2.43 (s, 3H), 2.10 (t, *J* = 12.4 Hz, 1H), 1.86 (td, *J* = 12.6, 4.4 Hz, 1H), 1.62 (d, *J* = 10.3 Hz, 1H); ^13^C NMR (101 MHz, CDCl_3_) *δ* 192.47, 152.72, 150.44, 148.12, 138.95, 134.22, 132.45, 130.97, 129.86, 129.37, 129.07, 128.71, 115.29, 114.74, 77.48, 77.16, 76.84, 56.28, 56.25, 55.06, 50.41, 46.46, 46.14, 42.91, 41.50, 37.13, 26.24; MS (ESI) *m*/*z* C_28_H_29_BrNO_5_^+^: [M + H]^+^_calcd_ =538.12236, and [M + H]^+^_measured_ =538.12183.

1-Br-4-(3-methyl)-cinnamic acid ester-sinomenine **2b:** white solid, 83% yield; m.p. 35–38 °C; ^1^H NMR (400 MHz, CDCl_3_) *δ* 8.18 (d, *J* = 16.0 Hz, 1H), 7.69–7.60 (m, 2H), 7.47 (dt, *J* = 25.4, 7.6 Hz, 2H), 6.86 (d, *J* = 16.0 Hz, 1H), 5.65 (d, *J* = 2.1 Hz, 1H), 4.12 (d, *J* = 15.8 Hz, 1H), 3.91 (s, 3H), 3.69 (s, 3H), 3.43 (t, *J* = 4.7 Hz, 1H), 3.30–3.15 (m, 2H), 2.75–2.66 (m, 3H), 2.60 (d, *J* = 17.4 Hz, 6H), 2.29 (t, *J* = 11.7 Hz, 1H), 2.04 (td, *J* = 12.5, 4.5 Hz, 1H); ^13^C NMR (101 MHz, CDCl_3_) *δ* 192.50, 164.60, 152.70, 150.45, 148.33, 138.97, 138.76, 134.14, 132.43, 131.81, 129.34, 129.01, 128.94, 125.88, 121.16, 116.37, 115.28, 114.74, 77.48, 77.16, 76.84, 56.27, 55.05, 50.38, 46.46, 46.12, 42.90, 41.48, 37.10, 26.22, 21.41; MS (ESI) *m*/*z* C_29_H_31_BrNO_5_^+^: [M + H]^+^_calcd_ = 552.13801, and [M + H]^+^_measured_ = 552.13721.

1-Br-4-(3-methoxy)-cinnamic acid ester-sinomenine **2c:** white solid, 81% yield; m.p. 35–38 °C; ^1^H NMR (400 MHz, CDCl_3_) *δ* 7.97 (dd, *J* = 16.2, 5.4 Hz, 1H), 7.63 (s, 1H), 7.52 (d, *J* = 7.3 Hz, 1H), 7.41–7.30 (m, 2H), 7.07 (s, 1H), 6.67 (d, *J* = 16.1 Hz, 1H), 5.46 (d, *J* = 2.1 Hz, 1H), 3.86 (s, 1H), 3.72 (s, 3H), 3.50 (s, 3H), 3.24 (t, *J* = 4.6 Hz, 1H), 3.09–2.96 (m, 2H), 2.51 (dd, *J* = 14.8, 4.5 Hz, 3H), 2.43 (s, 3H), 2.10 (t, *J* = 12.2 Hz, 1H), 1.87 (dt, *J* = 15.2, 7.5 Hz, 1H), 1.61 (s, 1H); ^13^C NMR (101 MHz, CDCl_3_) *δ* 192.47, 164.07, 152.76, 150.35, 138.83, 136.05, 135.14, 132.42, 130.78, 130.31, 130.07, 129.08, 128.45, 126.78, 115.33, 114.77, 77.48, 77.16, 76.84, 56.29, 56.26, 55.53, 55.07, 50.51, 50.36, 46.48, 46.42, 46.15, 42.91, 41.54, 37.16, and 26.25; MS (ESI) *m*/*z* C_29_H_31_BrNO_6_^+^: [M + H]^+^_calcd_ = 568.13292, and [M + H]^+^_measured_ = 568.13202.

1-Br-4-(3-Cl)-cinnamic acid ester-sinomenine **2d:** white solid, 77% yield; m.p. 35–38 °C; ^1^H NMR (400 MHz, CDCl_3_) *δ* 7.96 (d, *J* = 16.1 Hz, 1H), 7.63 (s, 1H), 7.52 (d, *J* = 7.1 Hz, 1H), 7.41–7.34 (m, 2H), 7.07 (s, 1H), 6.67 (d, *J* = 16.0 Hz, 1H), 5.46 (d, *J* = 2.1 Hz, 1H), 3.88 (d, *J* = 15.8 Hz, 1H), 3.72 (s, 3H), 3.50 (s, 3H), 3.24 (t, *J* = 4.6 Hz, 1H), 3.09–2.96 (m, 2H), 2.55–2.51 (m, 1H), 2.51–2.46 (m, 2H), 2.43 (s, 3H), 2.06 (s, 1H), 1.86 (td, *J* = 12.6, 4.6 Hz, 1H), 1.59 (d, *J* = 12.7 Hz, 1H); ^13^C NMR (101 MHz, CDCl_3_) *δ* 192.50, 152.72, 150.43, 148.09, 146.50, 135.55, 135.12, 130.79, 130.32, 130.07, 128.45, 126.78, 121.55, 118.13, 117.36, 116.87, 115.29, 114.76, 77.48, 77.16, 76.84, 56.28, 55.53, 55.07, 50.50, 50.36, 46.47, 46.11, 42.92, 41.49, 37.11, 26.23; MS (ESI) *m*/*z* C_28_H_28_BrClNO_5_^+^: [M + H]^+^_calcd_ = 572.08338, and [M + H]^+^_measured_ = 572.08240.

1-Br-4-(3-nitro)-cinnamic acid ester-sinomenine **2e:** yellow solid, 79% yield; m.p. 35–38 °C, ^1^H NMR (400 MHz, CDCl_3_) *δ* 8.49 (t, *J* = 1.9 Hz, 1H), 8.27 (dd, *J* = 8.2, 2.1 Hz, 1H), 8.08 (d, *J* = 16.0 Hz, 1H), 7.97 (d, *J* = 7.7 Hz, 1H), 7.62 (t, *J* = 8.0 Hz, 1H), 7.08 (s, 1H), 6.79 (d, *J* = 16.0 Hz, 1H), 5.48 (d, *J* = 2.1 Hz, 1H), 3.86 (d, *J* = 15.6 Hz, 1H), 3.73 (s, 3H), 3.51 (s, 3H), 3.25 (t, *J* = 4.7 Hz, 1H), 3.13–2.93 (m, 2H), 2.57–2.46 (m, 3H), 2.44 (s, 3H), 2.10 (t, *J* = 12.2 Hz, 1H), 1.88 (td, *J* = 12.6, 4.6 Hz, 1H), 1.61 (s, 1H); ^13^C NMR (101 MHz, CDCl_3_) *δ* 192.53, 163.62, 152.77, 150.24, 148.84, 145.14, 138.70, 136.01, 134.24, 132.37, 130.16, 129.15, 125.11, 122.98, 121.42, 119.92, 115.37, 114.87, 77.48, 77.16, 76.84, 56.29, 56.22, 55.09, 50.59, 46.37, 46.15, 42.90, 41.58, 37.19, 26.25; MS (ESI) *m*/*z* C_28_H_28_BrN_2_O_7_^+^: [M + H]^+^_calcd_ = 583.10744, and [M + H]^+^_measured_ = 583.10669.

### 2.2. Molecular Docking Test

The results of molecular docking are shown in Table 1. Using MOE software, the binding energy of the GBP5 protein to sinomenine was −6.6513 kcal/mol. Five derivatives were docked with GBP5, and their binding energy was less than that of SIN.

GBP5 is a potential target for sinomenine in inflammatory cells. Sinomenine directly binds to GBP5, inhibits its activity, regulates P2X7R, and inhibits downstream NLRP3-induced inflammatory pathways. Molecular docking showed that sinomenine could bind to the active pocket of GBP5 with a strong binding energy of −7.72 kcal/mol [20,21]. The **2a**–**2e** were docked with GBP5 protein (PDB no. 7ckf) by the molecular docking software MOE, with the binding site of GDP set as the docking pocket and one strand of the protein retained for docking. The docking results showed that **2c** has the lowest binding score, indicating that the energy required for binding to the protein is low and it is easier to bind. The highest docking score of **2a** is −7.61 kcal/mol, which is also relatively stable compared to the −6.65 kcal/mol of SIN. Meanwhile, **2a**–**2e** have a more stable binding with GBP5.

**Figure 1:**
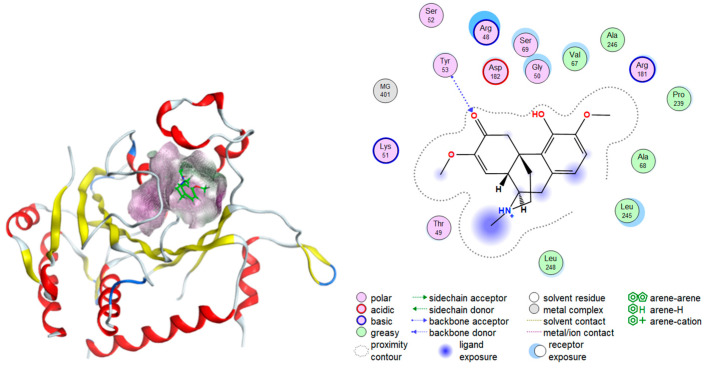
Molecular docking diagram of SIN.

The binding mode between the molecule and the target protein was analyzed using the MOE software. The molecular docking diagram of SIN is shown in Figure 1.

### 2.3. Druggability Analysis

ADMET prediction refers to the prediction of a series of key characteristics of drugs in vivo. The predicted LogP of SIN was 0.67, the F50% was between 0.7 and 0.9, the plasma protein binding was 39.2%, the blood–brain barrier was between 0.1 and 0.3, The HLM stability was between 0.5 and 0.7, the T_1/2_ was 3.604, the HH was 0.583 and the DIN was 0.255. Compared with SIN, the fat solubility increased, and the logP of each derivative increased. F50% decreased by 0.2–0.8. The plasma protein binding increased by 39.2–58.4% compared to SIN, of which **2a**, **2b**, **2c**, **2d**, **2e** were 96%, 96.5%, 95.7%, 97.3%, 97.6%, respectively, and the plasma protein binding rate was relatively high. The blood–brain barrier of **2a**, **2b**, **2d** increased by 0.4–0.9 compared with SIN, demonstrating that the permeability of the blood–brain barrier was relatively high. The HLM stability was 0.2–0.7 lower than that of SIN, and the probability of each derivative being metabolized in the liver was more stable than that of SIN. The T_1/2_ of **2a**–**2e** decreased by 1.72–2.29 compared to SIN, which was shorter half-life in the body. The HH of **2a**–**2e** was 0.04–0.10 higher than that of SIN, indicating that the derivatives had certain hepatotoxicity, unlike SIN. The DIN of **2a**–**2e** was 0.088–0.346 higher than that of SIN, indicating that the SIN derivatives had certain nephrotoxicity, unlike SIN. The results of druggability analysis are shown in Table 2.

### 2.4. Hot Plate Method

The pain threshold before high-dose administration of **2a** was 7.59 ± 1.67, and it was 9.92 ± 2.66 at 15 min. The pain threshold before high-dose administration of **2b** was 7.2 ± 2.19, and it was 10.30 ± 2.51 at 15 min and 9.67 ± 3.19 at 30 min. The pain threshold before the high-dose administration of **2c** was 8.72 ± 1.86, and it was 9.92 ± 2.66 at 30 min and 10.29 ± 1.57 at 60 min. The pain threshold before the high-dose administration of **2d** was 8.30 ± 2.71, and it was 10.88 ± 3.10 at 15 min. The pain threshold before the high-dose administration of **2e** was 8.36 ± 1.30, and it was 11.17 ± 2.53 at 15 min. The hot plate results show that the pain threshold of **2a**–**2e** was significantly increased compared to that before administration and compared to the saline group, indicating that it may have a significant analgesic effect. The results of the hot plate method for SIN and SIN derivatives are shown in Table 3.

### 2.5. Acetic Acid-Induced Writhing Test

The results of the acetic acid-induced writhing test are shown in Table 4. At the dose of 30 mg/kg, the writhing latency at each dose of **2a** was longer than the saline group and the inhibition rates of **2a** showed advantages compared to the SIN group. The inhibition rate of the writhing times of **2c** was higher in the high-dose group compared to the SIN group. The writhing latency of the high-dose **2e** was longer than the saline group and the SIN group. The inhibition rate of the medium and high dose **2e** was higher compared to the SIN group. The reason why the latency of writhing, the inhibition rate of writhing times, and the percentage of analgesia in each dose of the same derivative were significantly different may be related to the individual differences in mice.

### 2.6. Evaluation of Anti-Inflammatory Capacity in Mouse Ear Edema Model

In Table 5, the auricular swelling degree of **2b** was 0.0040 ± 0.0016 after stimulating the xylene, which was different from the saline group. The swelling inhibition rate of the mouse given **2b** was 35.48%. It was shown that **2b** could alleviate the inflammatory reaction caused by xylene. However, **2a**, **2c**, **2d**, **2e** had no significant difference from the saline group and SIN group.

## 3. Materials and Methods

### 3.1. Materials and Reagents

Sinomenine, pure 98%, Lot No. C15533222, Shanghai McLean Biochemical Technology Co. Ltd., Shanghai, China; p-Bromobenzoic acid, AR, Lot No.Lf1025177438, Shanghai Haohong Bio-medical Technology Co. Ltd., Shanghai, China; Dimethoxypyridine, AR, Lot No. RH626903, Shanghai Een Chemical Technology Co. Ltd., Shanghai, China; all other reagents were obtained from Shanghai Titan Technology Co, Shanghai, China, AR. SPF grade Kunming mice [SCXK (Xiang) 2019-0011], female, body mass (20 ± 4 g), Hunan Slike Jingda Laboratory Animal Co., Ltd., Changsha, China.

### 3.2. Synthesis and Chemical Characterization

SIN (6.0 mmol, 2 g) was dissolved in 20 mL of CH_2_Cl_2_, and N-bromosuccinimide (NBS) (6.66 mmol, 1.2 g) was dissolved in CH_2_Cl_2_ and slowly added to sinomenine. After stirring at room temperature for 12 h, saturated salt water was added. The water layer was extracted with CH_2_Cl_2_ (20 mL × 3), dried with anhydrous sodium sulfate, and the organic layer was merged, which was concentrated in vacuum. The residuals were purified by silica gel column of CH_2_Cl_2_ and CH_3_OH (30:1) to obtain the key intermediate 1 [22].

Subsequently, 1 (0. 6 g, 1.47 mmol), cinnamic acid (0.22 g, 2.21 mmol), EDC (0.46 g, 2.94 mmol), DMAP (0.27 g, 2.21 mmol), and 10 mL CH_2_Cl_2_ were stirred at room temperature for 10 h, and saturated salt water (3 × 10 mL) was added for extraction [23]. The organic layer was dried over by Na_2_SO_4_ and concentrated in vacuum. The residuals were purified by silica gel column of CH_2_Cl_2_ and CH_3_OH (50:1) to yield 1-Br-4-hydroxy ester derivatives **2a**–**2e**; the yields were above 77% (Scheme 1). All structures of these compounds were confirmed by ESI-MS, ^1^H NMR, and ^13^C NMR.

^1^H and ^13^C-NMR spectra were run on a Bruker Avance 400 MHz NMR spectrometer using tetramethylsilane (TMS) as the internal standard and CDCl_3_ as the solvent (Chemical shifts in δ, ppm). Splitting patterns were designated as follows: s—singlet; d—doublet; m—multiplet. The mass spectra were performed at the Q-Exactive Orbitrap MS (Thermo Fisher Scientific, Bremen, Germany) equipped with an electrospray ionization source.

### 3.3. Molecular Docking Test

Molecular docking studies were conducted to predict the binding affinity and interaction modes of sinomenine and its derivatives (**2a**–**2e**) with the guanylate-binding protein 5 (GBP5), a reported inflammatory regulator (PDB ID: 7CKF). Docking was performed using a Molecular Operating Environment (MOE, version 2019, Chemical Computing Group, Montreal, Canada) with the following validated protocol. The crystal structure was downloaded from the RCSB Protein Data Bank. All co-crystallized ligands, water molecules, and ions were removed. Hydrogen atoms were added, protonation states were assigned at physiological pH (7.4), and the protein was energy-minimized using the AMBER10:EHT force field to relieve steric clashes. The 2D structures of SIN and its derivatives were drawn in ChemDraw (version 2019) and converted into 3D geometries using MOE (version 2019). Ligands were protonated at pH 7.4, energy-minimized with the MMFF94x force field, and multiple low-energy conformations were generated (up to 30 per ligand). The binding site was defined around the co-crystallized GDP binding pocket, which has been implicated in GBP5 regulation. The Triangle Matcher algorithm was employed for pose generation, and the London dG scoring function was used for initial ranking. The top 30 poses were retained for each ligand, refined using force field-based minimization, and rescored with GBVI/WSA dG to estimate binding free energies. The best-ranked poses were selected for analysis. To assess docking reliability, the co-crystallized GDP ligand was re-docked into the binding pocket. The RMSD between the experimental and predicted binding conformations was 1.62 Å, indicating good agreement and validating the docking protocol.

Overall, these docking results suggest that the cinnamic acid esterification of sinomenine improves the binding affinity to GBP5, potentially enhancing its inhibitory effect on GBP5-mediated inflammatory signaling pathways.

### 3.4. Druggability Analysis

A Tencent AI Lab-iDrug platform was applied to predict the absorption distribution metabolism and drug formation of **2a**–**2e** in vivo.

### 3.5. Bioassay for In Vivo Analgesic and Anti-Inflammatory Activity

#### 3.5.1. Hot Plate Method

The mice were placed on a hot plate with a cut-off time of 30 s to avoid damaging the claws of the animals. The mice were randomly divided into 30 groups, 10 mice in each group. The mice in the blank group were intragastrically administered the same dose of normal saline, the SIN group was intragastrically administered 15 mg/kg of SIN, and the experimental group was intragastrically administered various doses of SIN derivatives (7.5 mg/kg in the low dose group, 15 mg/kg in the middle dose group, and 30 mg/kg in the high dose group). Gavage was applied for 3 days. After the last administration, the mice were recorded at 15 min, 30 min, 60 min intervals for licking the hind foot, as the reaction time (pain threshold) [24].

#### 3.5.2. Acetic Acid-Induced Writhing Test

Young Kunming mice were intraperitoneally injected with 0.6% acetic acid at a dose of 0.1 mL/kg before administration (10 mice in each group) [25]. The first writhing time (latency period) was recorded. Then, the sinomenine (15 mg/kg), normal saline (10 mL/kg) and various doses of SIN derivatives (7.5 mg/kg in the low dose group, 15 mg/kg in the middle dose group, and 30 mg/kg in the high dose group) were administered orally 3 days (once a day) prior to treatment with acetic acid. Half an hour after the last administration, the mice were intraperitoneally injected with 0.6% acetic acid. The first writhing time and the number of writhings in 15 min were observed and recorded immediately.

(1) $$\mathrm{The}\;\mathrm{prolonged}\;\mathrm{rate}\;\mathrm{of}\;\mathrm{latency}\;(\%)=\frac{\mathrm{time}\;\mathrm{after}\;\mathrm{administration}-\mathrm{time}\;\mathrm{before}\;\mathrm{administration}}{\mathrm{time}\;\mathrm{before}\;\mathrm{administration}}\;\times \;100\%$$

(2) $$\mathrm{Percentage}\;\mathrm{of}\;\mathrm{analgesia}\;(\%)=\frac{\;\mathrm{number}\;\mathrm{of}\;\mathrm{twisted}\;\mathrm{rats}\;\mathrm{in}\;\mathrm{control}\;\mathrm{group}-\mathrm{number}\;\mathrm{of}\;\mathrm{twisted}\;\mathrm{rats}\;\mathrm{in}\;\mathrm{experimental}\;\mathrm{group}}{numberoftwistedratsincontrolgroup}\;\times \;100\%$$

(3) $$\mathrm{Writhing}\;\mathrm{times}\;\mathrm{inhibition}\;\mathrm{rate}\;\left(\%\right)=\;\frac{\mathrm{mean}\;\mathrm{number}\;\mathrm{of}\;\mathrm{body}\;\mathrm{torsions}\;\mathrm{in}\;\mathrm{the}\;\mathrm{control}\;\mathrm{group}-\mathrm{mean}\;\mathrm{number}\;\mathrm{of}\;\mathrm{body}\;\mathrm{torsions}\;\mathrm{in}\;\mathrm{the}\;\mathrm{experimental}\;\mathrm{group}}{\mathrm{mean}\;\mathrm{number}\;\mathrm{of}\;\mathrm{body}\;\mathrm{torsions}\;\mathrm{in}\;\mathrm{the}\;\mathrm{control}\;\mathrm{group}}\;\times \;100\%$$

#### 3.5.3. Evaluation of Anti-Inflammatory Capacity in Mouse Ear Edema Model

Thirty young male Kunming mice (20–25 g) were classified into the following three groups at random. The three groups are the experimental group, the positive control group, and the blank group. The experimental group were given sinomenine 4-cinnamic acid ester derivatives at 30 mg/kg. The positive control sample received 30 mg/kg SIN. The blank sample were given saline. Each sample was treated with the medicine for 7 days continuously. A total of 30 min after the last administration, 40 μL of xylene was evenly coated on the two sides of the mouse [26].

The mice were cervically dislocated and killed 40 min after xylene was applied, and an 8 mm round piece of ear was taken at the same position in the right and left ears using a perforator and weighed with a precision analytical balance. The absolute values of the differences in mass and thickness between the two ears were taken as the degree of ear edema.

### 3.6. Statistical Analysis

Results were analyzed using SPSS 20.0. Multiple comparisons were performed using one-way ANOVA followed by an LSD *t*-test. Results were considered statistically significant with a *p* < 0.05. Results are expressed as mean ± SD.

## 4. Conclusions

In conclusion, sinomenine 1-Br-4-cinnamic acid ester derivatives were highly selectively synthesized. The reaction occurred readily with sinomenine and cinnamic acid. The yields ranged from good to excellent. The structures of the target compounds were established by ^1^H NMR, ^13^C NMR, and MS spectra. It may be widely used for modifying these polyfunctional natural products.

Five derivatives had good binding activity with the GBP5 protein by molecular docking software MOE. Pre-preparing the druggability of the derivatives by ADEMT 3.0 showed that each derivative had similar druggability to SIN. The analgesic activities of five derivatives were evaluated by the hot plate method and the acetic acid writhing method. The results showed that the analgesic effects of **2a**, **2c**, **2e** were better than that of sinomenine, and the other derivatives were equivalent to sinomenine. Furthermore, the auricular swelling degree of **2b** was lower than SIN and the swelling percentages of **2b** were higher than SIN in the xylene-induced ear edema assay. Therefore, **2b** was also confirmed to have a potent anti-inflammatory effect. This research established a basis theory for the development and application of 1-Br-4-cinnamic acid ester derivatives as natural analgesic and anti-inflammatory agents. Altogether, the results related to the series of SIN derivatives support the potential value of analgesia and its anti-inflammatory properties and have practical significance for the expansion of the clinical applications of SIN.

## Figures and Tables

**Table 1:** Molecular docking score of sinomenine and sinomenine derivatives.

Model	Binding Energy (kcal/mol)
SIN	−6.65
**2a**	−7.61
**2b**	−7.17
**2c**	−9.31
**2d**	−7.54
**2e**	−8.57

**Table 2:** Analysis of druggability of SIN and SIN derivatives.

Drug	logP	F50%	PPB	BBB	HLM Stability	T_1/2_	HH	DIN
SIN	0.67	0.7–0.9	39.2%	0.1–0.3	0.5–0.7	3.60	0.583	0.255
**2a**	3.75	0.3–0.5	96.0%	0.7–0.9	0–0.1	1.88	0.675	0.425
**2b**	4.01	0.3–0.5	96.5%	0.7–0.9	0.1–0.3	1.52	0.685	0.448
**2c**	3.78	0.5–0.7	95.7%	0.1–0.3	0–0.1	1.70	0.621	0.524
**2d**	4.06	0.3–0.5	97.3%	0.9–1.0	0–0.1	1.31	0.685	0.621
**2e**	3.57	0.1–0.3	97.6%	0.1–0.3	0–0.1	1.57	0.676	0.343

**Table 3:** Results of the hot plate method for SIN and SIN derivatives.

Compounds	Dose (mg/kg)	Pain Threshold Before Administration $\overset{-}{X}$ ± s (s)	Pain Threshold at Different Times After Administration (s)		
			15 min	30 min	60 min
SIN	15	9.91 ± 2.62	10.39 ± 3.26 ^▲^	11.63 ± 2.36 *^▲^	10.72 ± 3.07
**2a_C1_**	30	7.59 ± 1.67	9.92 ± 2.66 *^▲^	9.26 ± 3.19 ^Δ^	8.85 ± 2.08 *
**2a_C2_**	15	8.34 ± 3.14	8.33 ± 1.87	6.88 ± 3.17 *^Δ^	8.15 ± 2.49 ^Δ^
**2a_C3_**	7.5	7.62 ± 2.35	8.74 ± 3.46	9.96 ± 2.79 ^▲^	8.42 ± 3.26
**2b_C1_**	30	7.25 ± 2.19	10.30 ± 2.51 *^▲^	9.67 ± 3.19 *^▲^	7.66 ± 2.95 ^Δ^
**2b_C2_**	15	6.36 ± 1.24	7.63 ± 1.43 *^Δ^	6.57 ± 1.61 ^Δ^	7.18 ± 2.25 ^Δ^
**2b_C3_**	7.5	6.10 ± 1.04	7.52 ± 2.51 *^Δ^	7.92 ± 2.19 *^Δ^	6.97 ± 2.20 ^Δ^
**2c_C1_**	30	8.72 ± 1.86	8.05 ± 2.27	9.85 ± 1.20 *^▲Δ^	10.29 ± 1.57 *^▲^
**2c_C2_**	15	8.40 ± 2.44	7.85 ± 1.85 ^Δ^	7.63 ± 1.22 ^Δ^	7.39 ± 0.68 ^Δ^
**2c_C3_**	7.5	7.72 ± 1.30	7.77 ± 1.52 ^Δ^	8.76 ± 1.62 ^Δ^	6.71 ± 2.24 ^Δ^
**2d_C1_**	30	8.30 ± 2.71	10.88 ± 3.10 *^▲^	8.17 ± 3.60 ^Δ^	10.07 ± 3.57 *
**2d_C2_**	15	6.40 ± 2.76	7.67 ± 3.02 *	7.57 ± 2.00 ^Δ^	9.21 ± 2.75 *
**2d_C3_**	7.5	8.20 ± 2.05	9.31 ± 2.58	8.36 ± 2.56 ^Δ^	8.72 ± 1.79
**2e_C1_**	30	8.36 ± 1.30	11.17 ± 2.53 *^▲^	8.26 ± 2.12 ^Δ^	10.96 ± 3.48 ^▲^
**2e_C2_**	15	7.50 ± 2.80	9.30 ± 2.52	9.19 ± 4.32	10.26 ± 3.63 *
**2e_C3_**	7.5	6.56 ± 0.99	7.43 ± 2.09 ^Δ^	7.65 ± 2.56 ^Δ^	8.17 ± 2.48 ^Δ^
saline		7.70 ± 1.56	7.53 ± 1.59	7.07 ± 1.65	8.26 ± 1.79

*, *p* < 0.05 compared with the pain threshold before administration; ▲, *p* < 0.05 compared with saline; Δ, *p* < 0.05 compared with the sinomenine group. Results are expressed as means ± SD (n = 10 per group).

**Table 4:** Results of acetic acid-induced writhing test for sinomenine and sinomenine derivatives.

Compounds	Dose (mg/kg)	The Prolonged Rate of Latency (%)	Writhing Times Inhibition Rate (%)	Percentage of Analgesia (%)
SIN	15	39.10	29.47	0
**2a_C1_**	30	31.33	43.72	0
**2a_C2_**	15	32.25	36.59	0
**2a_C3_**	7.5	24.99	46.85	0
**2b_C1_**	30	35.50	33.07	0
**2b_C2_**	15	33.05	25.12	0
**2b_C3_**	7.5	17.64	18.36	0
**2c_C1_**	30	11.44	57.13	0
**2c_C2_**	15	27.77	29.95	0
**2c_C3_**	7.5	38.31	28.14	0
**2d_C1_**	30	11.56	35.27	0
**2d_C2_**	15	23.08	39.61	0
**2d_C3_**	7.5	28.51	37.20	0
**2e_C1_**	30	55.56	57.00	0
**2e_C2_**	15	20.85	45.65	0
**2e_C3_**	7.5	19.79	24.64	0
saline		18.10		0

**Table 5:** Results of the mouse ear swelling experiment.

Compounds	Dose (mg/kg)	Auricular Swelling Degree (X ± s)	Swelling Inhibition Rate (%)
SIN	30	0.0043 ± 0.0016 ^▲^	32.26
**2a**	30	0.0049 ± 0.0018	20.98
**2b**	30	0.0040 ± 0.0016 ^▲^	35.48
**2c**	30	0.0047 ± 0.0026	24.19
**2d**	30	0.0061 ± 0.0028	1.61
**2e**	30	0.0044 ± 0.0017 ^▲^	29.03
saline		0.0062 ± 0.0024 ^Δ^	

▲, Compared with the saline group, *p* < 0.05; Δ, compared with the SIN group, *p* < 0.05.

## Data Availability

Data are contained within the article and Supplementary Materials.

## Supplementary Materials

The following supporting information can be downloaded at https://www.mdpi.com/article/10.3390/molecules30183802/s1, Figures S1–S6 correspond to ADMET prediction and druggability radar chart Figures S7–S11. NMR Spectra Figures S12–S21. The HRMS figures correspond to Figures S22–S26.
