# Supplementary material for: Semi-Synthesis and Biological Evaluation of Novel Sinomenine Derivatives
Source: Molecules. 2025 Sep 18;30(18):3802. doi: 10.3390/molecules30183802 (PMC12472971; doi:10.3390/molecules30183802)
Supplement: Supplementary file 1 [file molecules-30-03802-s001.zip › molecules-3841216-supplementary.pdf]

Supplementary Materials.

Semi-Synthesis and Biological Evaluation of Novel Sinomenine Derivatives

Meichun Wu [1,2,3], Zhewei Zhang [1,2,3], Ze Li [1,2,3] and Zijian Zhao [1,2,3,*]

[1] College of Chemistry and Materials Engineering, Huaihua University, Huaihua 418000, China; cq0078@163.com (M.W.); zzw010718@163.com (Z.Z.); a187z3423788@163.com (Z.L.)
[2] Hunan Provincial Engineering Technology Research Center for Polyvinyl Alcohol Based New Functional Materials, Huaihua 418000, China
[3] Key Laboratory of Research and Utilization of Ethnomedicinal Plant Resources of Hunan Province, Huaihua University, Huaihua 418000, China
* Correspondence: zjzhao72@163.com (Z.Z.)

Index.

- **ADMET prediction (sinomenine and compounds 2a–2e, Figures S1–S6), pages 3-8.**
- **Druggability radar chart (compounds 2a–2e, Figures S7–S11), pages 9-13.**
- **Supplementary Figures ^{1}H-NMR Spectra (compounds 2a–2e, Figures S12–S16), pages 14-18.**
- **Supplementary Figures ^{13}C-NMR Spectra (compounds 2a-2e, Figures S17–S21), pages 19-23.**
- **High resolution mass spectrum (HRMS): (compounds 2a–2b, Figures S22–S26), pages 24-26.**

ADMET prediction (sinomenine and compounds 2a–2e, Figures S1–S6)

PHYSICOCHEMICAL PROPERTY

Molecular Weight (MW)	329.16
Volume	335.93
Density	0.98
nHA	5.0
nHD	1.0
nRot	2.0
nRing	4.0
MaxRing	12.0
nHet	5.0
fChar	0.0
nRig	21.0
Flexibility	0.095
Stereo Centers	3.0
TPSA	59.0
logS	-1.72
logP	0.672
logD7.4	0.735
pka (Acid)	9.558
pka (Base)	8.181
Melting point	168.297
Boiling point	284.876

ABSPORPTION

Caco-2 Permeability	-4.955
MDCK Permeability	-4.771
PAMPA	---
Pgp inhibitor	-
Pgp substrate	---
HIA	---
F20%	---
F30%	---
F50%	++

DISTRIBUTION

PPB	39.2%
VDss	0.189
BBB	--
Fu	64.6%
OATP1B1 inhibitor	+++
OATP1B3 inhibitor	+++
BCRP inhibitor	--
MRP1 inhibitor	++
BSEP inhibitor	-

METABOLISM

CYP1A2 inhibitor	---
CYP1A2 substrate	+++
CYP2C19 inhibitor	---
CYP2C19 substrate	+++
CYP2C9 inhibitor	---
CYP2C9 substrate	++
CYP2D6 inhibitor	---
CYP2D6 substrate	+++
CYP3A4 inhibitor	---
CYP3A4 substrate	+++
CYP2B6 inhibitor	---
CYP2B6 substrate	+++
CYP2C8 inhibitor	---
HLM Stability	+

EXCRETION

CL_{plasma}	8.144
T1/2	3.604

TOXICITY

hERG Blockers	0.185
hERG Blockers (10um)	0.524
DILI	0.13
AMES Toxicity	0.581
Rat Oral Acute Toxicity	0.702
FDAMDD	0.785
Skin Sensitization	0.418
Carcinogenicity	0.441
Eye Corrosion	0.0
Eye Irritation	0.046
Respiratory	0.848
Human Hepatotoxicity	0.583
Drug-induced Nephrotoxicity	0.255
Drug-induced Neurotoxicity	0.529
Ototoxicity	0.833
Hematotoxicity	0.182
Genotoxicity	0.854
RPMI-8226 Immunitoxicity	0.081
A549 Cytotoxicity	0.076
Hek293 Cytotoxicity	0.351
BCF	0.893
IGC50	3.634
LC50DM	4.954
LC50FM	4.386

Figure S1. ADMET prediction of sinomenine

PHYSICOCHEMICAL PROPERTY

Molecular Weight (MW)	537.12
Volume	497.929
Density	1.079
nHA	6.0
nHD	0.0
nRot	6.0
nRing	5.0
MaxRing	12.0
nHet	7.0
fChar	0.0
nRig	29.0
Flexibility	0.207
Stereo Centers	3.0

TPSA	65.07
logS	-4.289
logP	3.754
logD7.4	3.359
pka (Acid)	8.603
pka (Base)	6.792
Melting point	124.445
Boiling point	307.606

ABSPORPTION

Caco-2 Permeability	-5.045
MDCK Permeability	-4.622
PAMPA	---
Pgp inhibitor	+++
Pgp substrate	---
HIA	---
F20%	---
F30%	---
F50%	-

DISTRIBUTION

PPB	96.0%
VDss	0.422
BBB	++
Fu	3.3%
OATP1B1 inhibitor	+++
OATP1B3 inhibitor	+++
BCRP inhibitor	---
MRP1 inhibitor	++
BSEP inhibitor	+++

METABOLISM

CYP1A2 inhibitor	+++
CYP1A2 substrate	---
CYP2C19 inhibitor	+++
CYP2C19 substrate	---
CYP2C9 inhibitor	++
CYP2C9 substrate	---
CYP2D6 inhibitor	---
CYP2D6 substrate	+
CYP3A4 inhibitor	---
CYP3A4 substrate	---
CYP2B6 inhibitor	+++
CYP2B6 substrate	---
CYP2C8 inhibitor	+++
HLM Stability	---

EXCRETION

CL_{plasma}	8.634
T1/2	1.88

TOXICITY

hERG Blockers	0.156
hERG Blockers (10um)	0.682
DILI	0.785
AMES Toxicity	0.591
Rat Oral Acute Toxicity	0.876
FDAMDD	0.988
Skin Sensitization	0.971
Carcinogenicity	0.242
Eye Corrosion	0.0
Eye Irritation	0.004
Respiratory	0.868
Human Hepatotoxicity	0.675
Drug-induced Nephrotoxicity	0.425
Drug-induced Neurotoxicity	0.844
Ototoxicity	0.785
Hematotoxicity	0.073
Genotoxicity	0.989
RPMI-8226 Immunitoxicity	0.1
A549 Cytotoxicity	0.312
Hek293 Cytotoxicity	0.776
BCF	1.578
IGC50	4.741
LC50DM	6.297
LC50FM	5.901

Figure S2. ADMET prediction of 1-Br-4-cinnamic acid ester-sinomenine (2a)

PHYSICOCHEMICAL PROPERTY

Molecular Weight (MW)	551.13
Volume	515.225
Density	1.07
nHA	6.0
nHD	0.0
nRot	6.0
nRing	5.0
MaxRing	12.0
nHet	7.0
fChar	0.0
nRig	29.0
Flexibility	0.207
Stereo Centers	3.0
TPSA	65.07
logS	-4.343
logP	4.014
logD7.4	3.478
pka (Acid)	8.862
pka (Base)	6.975
Melting point	124.728
Boiling point	309.774

ABSPORPTION

Caco-2 Permeability	-5.044
MDCK Permeability	-4.598
PAMPA	---
Pgp inhibitor	+++
Pgp substrate	---
HIA	---
F20%	---
F30%	---
F50%	-

DISTRIBUTION

PPB	96.5%
VDss	0.408
BBB	++
Fu	2.7%
OATP1B1 inhibitor	+++
OATP1B3 inhibitor	+++
BCRP inhibitor	---
MRP1 inhibitor	+++
BSEP inhibitor	+++

TOXICITY

hERG Blockers	0.156
hERG Blockers (10um)	0.672
DILI	0.769
AMES Toxicity	0.566
Rat Oral Acute Toxicity	0.866
FDAMDD	0.988
Skin Sensitization	0.972
Carcinogenicity	0.246
Eye Corrosion	0.0
Eye Irritation	0.004
Respiratory	0.88
Human Hepatotoxicity	0.685
Drug-induced Nephrotoxicity	0.448
Drug-induced Neurotoxicity	0.852
Ototoxicity	0.816
Hematotoxicity	0.084
Genotoxicity	0.988
RPMI-8226 Immunitoxicity	0.117
A549 Cytotoxicity	0.333
Hek293 Cytotoxicity	0.721
BCF	1.53
IGC50	4.758
LC50DM	6.142
LC50FM	5.804

METABOLISM

CYP1A2 inhibitor	++
CYP1A2 substrate	--
CYP2C19 inhibitor	+++
CYP2C19 substrate	---
CYP2C9 inhibitor	--
CYP2C9 substrate	---
CYP2D6 inhibitor	---
CYP2D6 substrate	-
CYP3A4 inhibitor	--
CYP3A4 substrate	---
CYP2B6 inhibitor	+++
CYP2B6 substrate	---
CYP2C8 inhibitor	+++
HLM Stability	--

EXCRETION

CL_{plasma}	8.417
T1/2	1.524

Figure S3. ADMET prediction of 1-Br-4-(3-methyl)-cinnamic acid ester-sinomenine (2b)

PHYSICOCHEMICAL PROPERTY

Molecular Weight (MW)	567.13
Volume	524.015
Density	1.082
nHA	7.0
nHD	0.0
nRot	7.0
nRing	5.0
MaxRing	12.0
nHet	8.0
fChar	0.0
nRig	29.0
Flexibility	0.241
Stereo Centers	3.0

TPSA	74.3
logS	-4.516
logP	3.784
logD7.4	3.324
pka (Acid)	8.884
pka (Base)	6.702
Melting point	123.812
Boiling point	307.603

ABSORPTION

Caco-2 Permeability	-5.007
MDCK Permeability	-4.723
PAMPA	---
Pgp inhibitor	+++
Pgp substrate	---
HIA	---
F20%	---
F30%	---
F50%	+

DISTRIBUTION

PPB	95.7%
VDss	0.409
BBB	--
Fu	3.3%
OATP1B1 inhibitor	+++
OATP1B3 inhibitor	+++
BCRP inhibitor	--
MRP1 inhibitor	++
BSEP inhibitor	+++

TOXICITY

hERG Blockers	0.178
hERG Blockers (10um)	0.671
DILI	0.823
AMES Toxicity	0.619
Rat Oral Acute Toxicity	0.879
FDAMDD	0.988
Skin Sensitization	0.948
Carcinogenicity	0.282
Eye Corrosion	0.0
Eye Irritation	0.004
Respiratory	0.904
Human Hepatotoxicity	0.621
Drug-induced Nephrotoxicity	0.524
Drug-induced Neurotoxicity	0.851
Ototoxicity	0.823
Hematotoxicity	0.083
Genotoxicity	0.971
RPMI-8226 Immunitoxicity	0.129
A549 Cytotoxicity	0.367
Hek293 Cytotoxicity	0.77
BCF	1.559
IGC50	4.626
LC50DM	6.253
LC50FM	5.764

METABOLISM

CYP1A2 inhibitor	++
CYP1A2 substrate	--
CYP2C19 inhibitor	+++
CYP2C19 substrate	++
CYP2C9 inhibitor	+
CYP2C9 substrate	---
CYP2D6 inhibitor	---
CYP2D6 substrate	++
CYP3A4 inhibitor	--
CYP3A4 substrate	---
CYP2B6 inhibitor	+++
CYP2B6 substrate	---
CYP2C8 inhibitor	+++
HLM Stability	---

EXCRETION

CL_{plasma}	9.571
T1/2	1.701

Figure S4. ADMET prediction of 1-Br-4-(3-methoxy)-cinnamic acid ester-sinomenine (2c)

PHYSICOCHEMICAL PROPERTY

Molecular Weight (MW)	571.08
Volume	513.14
Density	1.113
nHA	6.0
nHD	0.0
nRot	6.0
nRing	5.0
MaxRing	12.0
nHet	8.0
fChar	0.0
nRig	29.0
Flexibility	0.207
Stereo Centers	3.0
TPSA	65.07
logS	-4.43
logP	4.063
logD7.4	3.54
pka (Acid)	8.91
pka (Base)	6.92
Melting point	129.284
Boiling point	316.119

ABSPORPTION

Caco-2 Permeability	-5.08
MDCK Permeability	-4.653
PAMPA	---
Pgp inhibitor	+++
Pgp substrate	---
HIA	---
F20%	---
F30%	---
F50%	--

DISTRIBUTION

PPB	97.3%
VDss	0.496
BBB	+++
Fu	2.2%
OATP1B1 inhibitor	+++
OATP1B3 inhibitor	+++
BCRP inhibitor	---
MRP1 inhibitor	+++
BSEP inhibitor	+++

TOXICITY

hERG Blockers	0.238
hERG Blockers (10um)	0.757
DILI	0.875
AMES Toxicity	0.468
Rat Oral Acute Toxicity	0.887
FDAMDD	0.989
Skin Sensitization	0.976
Carcinogenicity	0.215
Eye Corrosion	0.0
Eye Irritation	0.002
Respiratory	0.839
Human Hepatotoxicity	0.685
Drug-induced Nephrotoxicity	0.621
Drug-induced Neurotoxicity	0.876
Ototoxicity	0.844
Hematotoxicity	0.092
Genotoxicity	0.992
RPMI-8226 Immunitoxicity	0.11
A549 Cytotoxicity	0.538
Hek293 Cytotoxicity	0.868
BCF	1.726
IGC50	4.933
LC50DM	6.659
LC50FM	6.213

METABOLISM

CYP1A2 inhibitor	+++
CYP1A2 substrate	---
CYP2C19 inhibitor	+++
CYP2C19 substrate	---
CYP2C9 inhibitor	++
CYP2C9 substrate	---
CYP2D6 inhibitor	---
CYP2D6 substrate	++
CYP3A4 inhibitor	---
CYP3A4 substrate	---
CYP2B6 inhibitor	+++
CYP2B6 substrate	---
CYP2C8 inhibitor	+++
HLM Stability	---

EXCRETION

CL_{plasma}	6.849
T1/2	1.312

Figure S5. ADMET prediction of 1-Br-4-(3-Cl)-cinnamic acid ester-sinomenine (2d)

PHYSICOCHEMICAL PROPERTY

Molecular Weight (MW)	582.1
Volume	523.869
Density	1.111
nHA	9.0
nHD	0.0
nRot	7.0
nRing	5.0
MaxRing	12.0
nHet	10.0
fChar	0.0
nRig	30.0
Flexibility	0.233
Stereo Centers	3.0
TPSA	108.21
logS	-4.864
logP	3.57
logD7.4	3.159
pka (Acid)	9.103
pka (Base)	6.018
Melting point	145.332
Boiling point	316.724

ABSPORPTION

Caco-2 Permeability	-4.968
MDCK Permeability	-4.468
PAMPA	---
Pgp inhibitor	+++
Pgp substrate	---
HIA	---
F20%	---
F30%	---
F50%	--

DISTRIBUTION

PPB	97.6%
VDss	0.212
BBB	--
Fu	2.5%
OATP1B1 inhibitor	+++
OATP1B3 inhibitor	+++
BCRP inhibitor	---
MRP1 inhibitor	+++
BSEP inhibitor	+++

TOXICITY

hERG Blockers	0.133
hERG Blockers (10um)	0.682
DILI	0.983
AMES Toxicity	0.874
Rat Oral Acute Toxicity	0.94
FDAMDD	0.99
Skin Sensitization	0.996
Carcinogenicity	0.262
Eye Corrosion	0.0
Eye Irritation	0.011
Respiratory	0.932
Human Hepatotoxicity	0.676
Drug-induced Nephrotoxicity	0.343
Drug-induced Neurotoxicity	0.179
Ototoxicity	0.778
Hematotoxicity	0.112
Genotoxicity	1.0
RPMI-8226 Immunitoxicity	0.091
A549 Cytotoxicity	0.411
Hek293 Cytotoxicity	0.834
BCF	1.504
IGC50	4.674
LC50DM	6.177
LC50FM	5.811

METABOLISM

CYP1A2 inhibitor	+++
CYP1A2 substrate	---
CYP2C19 inhibitor	+++
CYP2C19 substrate	---
CYP2C9 inhibitor	--
CYP2C9 substrate	---
CYP2D6 inhibitor	---
CYP2D6 substrate	-
CYP3A4 inhibitor	--
CYP3A4 substrate	---
CYP2B6 inhibitor	+++
CYP2B6 substrate	---
CYP2C8 inhibitor	+++
HLM Stability	---

EXCRETION

CL_{plasma}	7.22
T1/2	1.566

Figure S6. ADMET prediction of 1-Br-4-(3-nitro)-cinnamic acid ester-sinomenine (2e)

Druggability radar chart (compounds 2a–2e, Figures S7–S11)

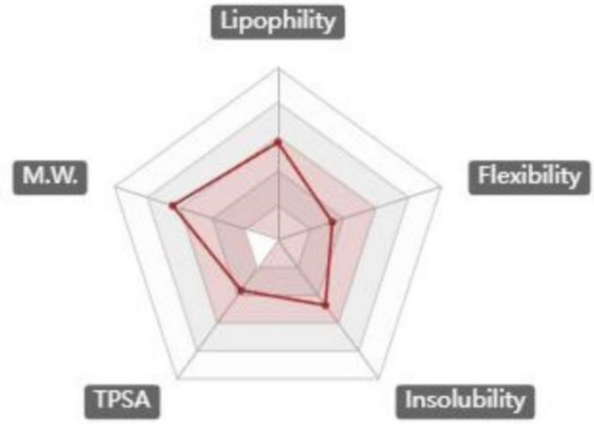

Figure S7. Druggability radar chart of 1-Br-4-cinnamic acid ester-sinomenine (2a)

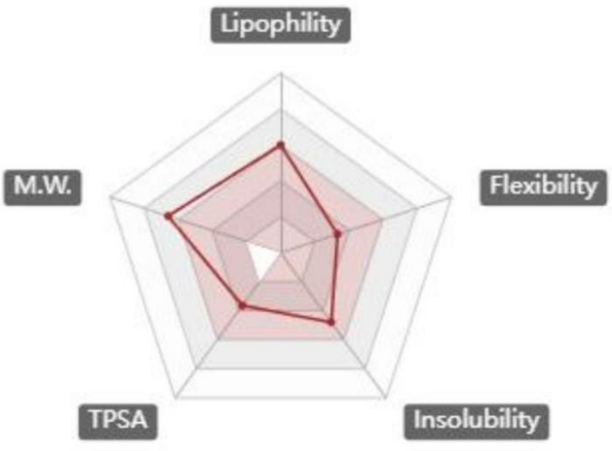

Figure S8. Druggability radar chart of 1-Br-4-(3-methyl)-cinnamic acid ester-sinomenine(2b)

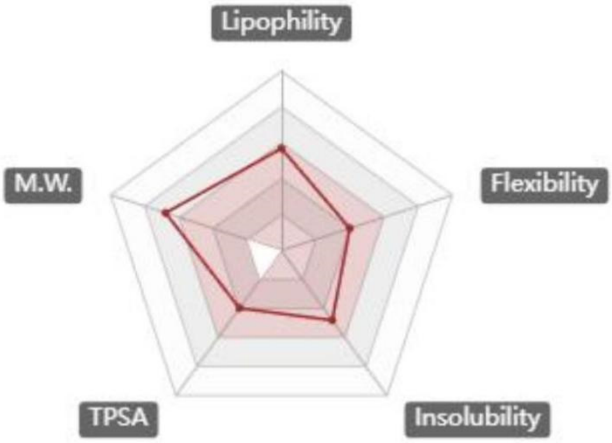

Figure S9. Druggability radar chart of 1-Br-4-(3-methoxy)-cinnamic acid ester-sinomenine (2c)

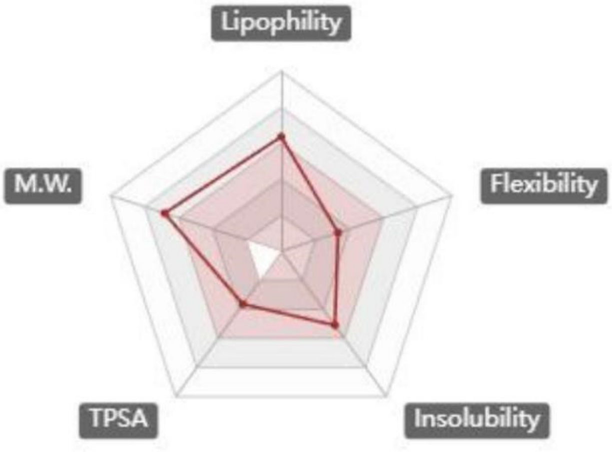

Figure S10. Druggability radar chart of 1-Br-4-(3-Cl)-cinnamic acid ester-sinomenine (2d)

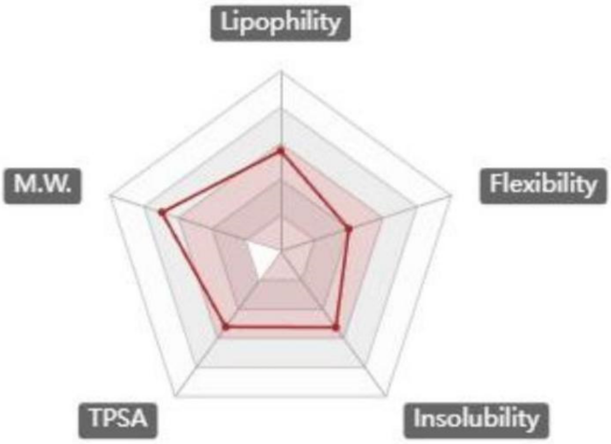

Figure S11. Druggability radar chart of 1-Br-4-(3-nitro)-cinnamic acid ester-sinomenine (2e)

Supplementary Figures ^{1}H-NMR Spectra (compounds 2a–2e, Figures S12–S16)

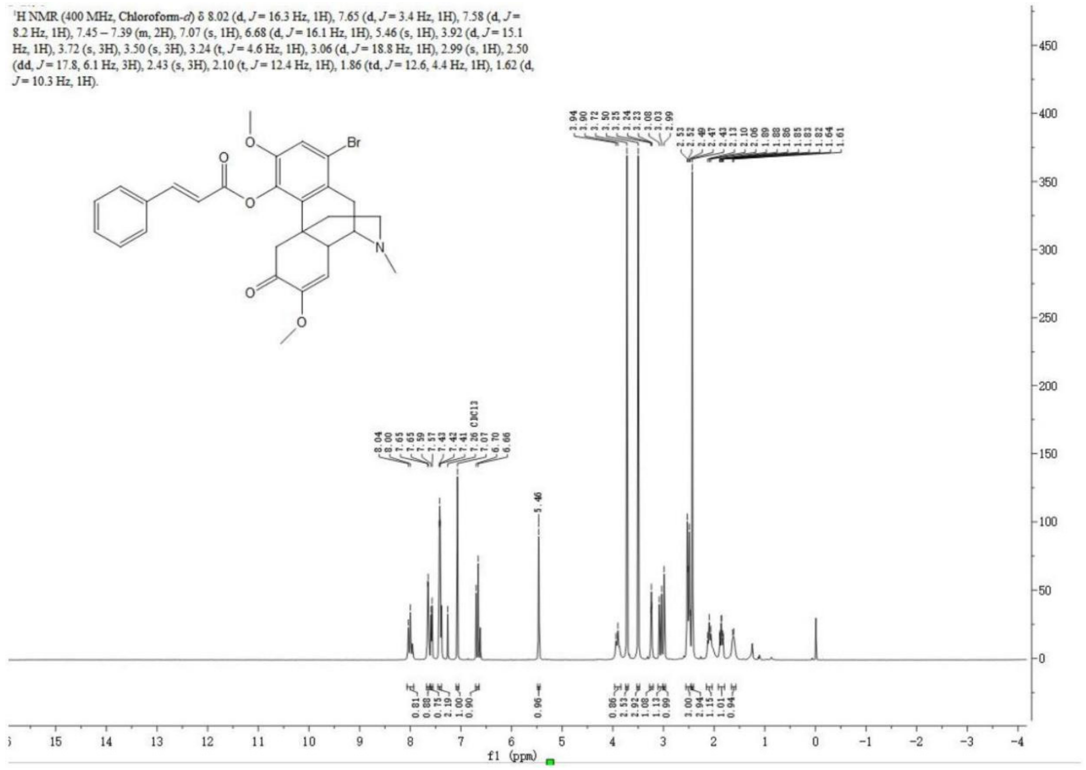

Figure S12. ^{1}H-NMR of1-Br-4-cinnamic acid ester-sinomenine (2a) (deuterated solvent used: $CDCl_3$)

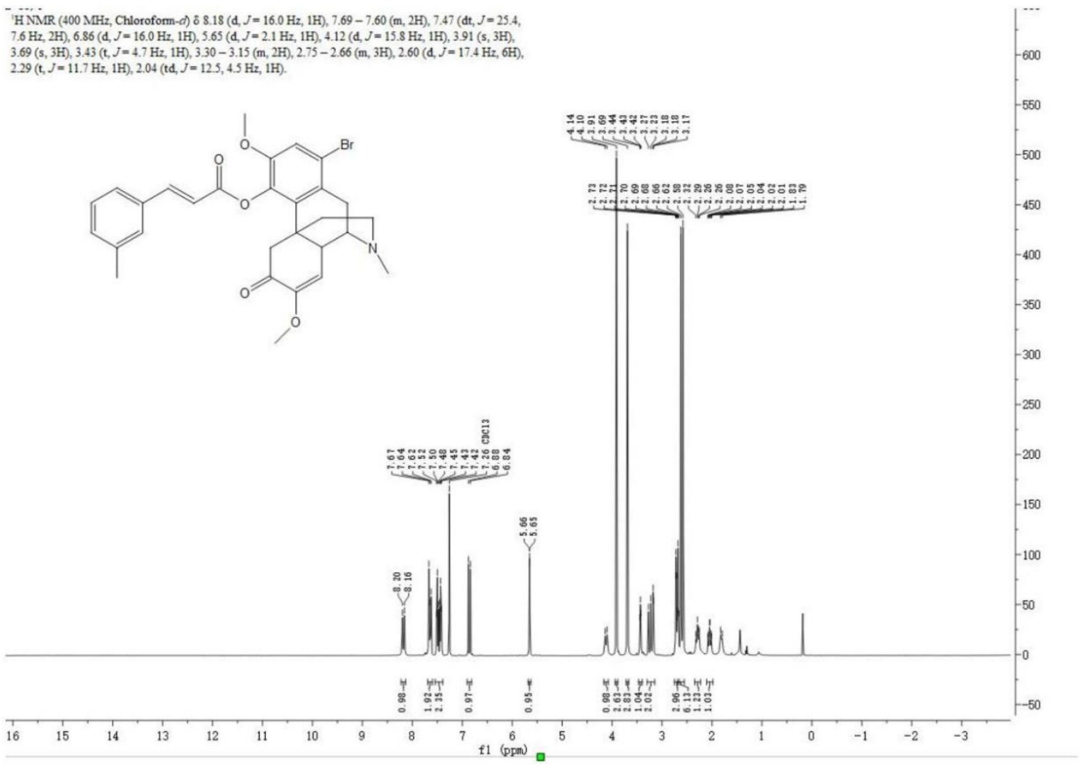

Figure S13. ¹H-NMR of 1-Br-4-(3-methyl)-cinnamic acid ester-sinomenine (2b) (deuterated solvent used: $CDCl_3$)

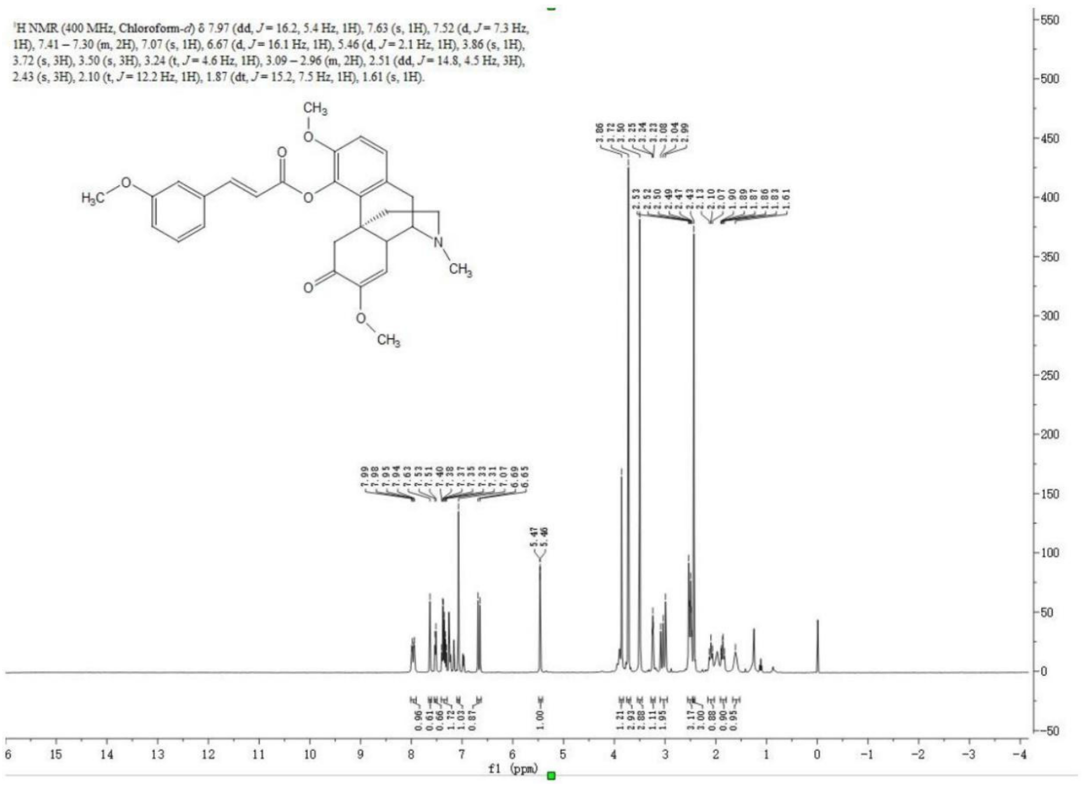

Figure S14. ^{1}H-NMR of 1-Br-4-(3-methoxy)-cinnamic acid ester-sinomenine (2c) (deuterated solvent used: $CDCl_3$)

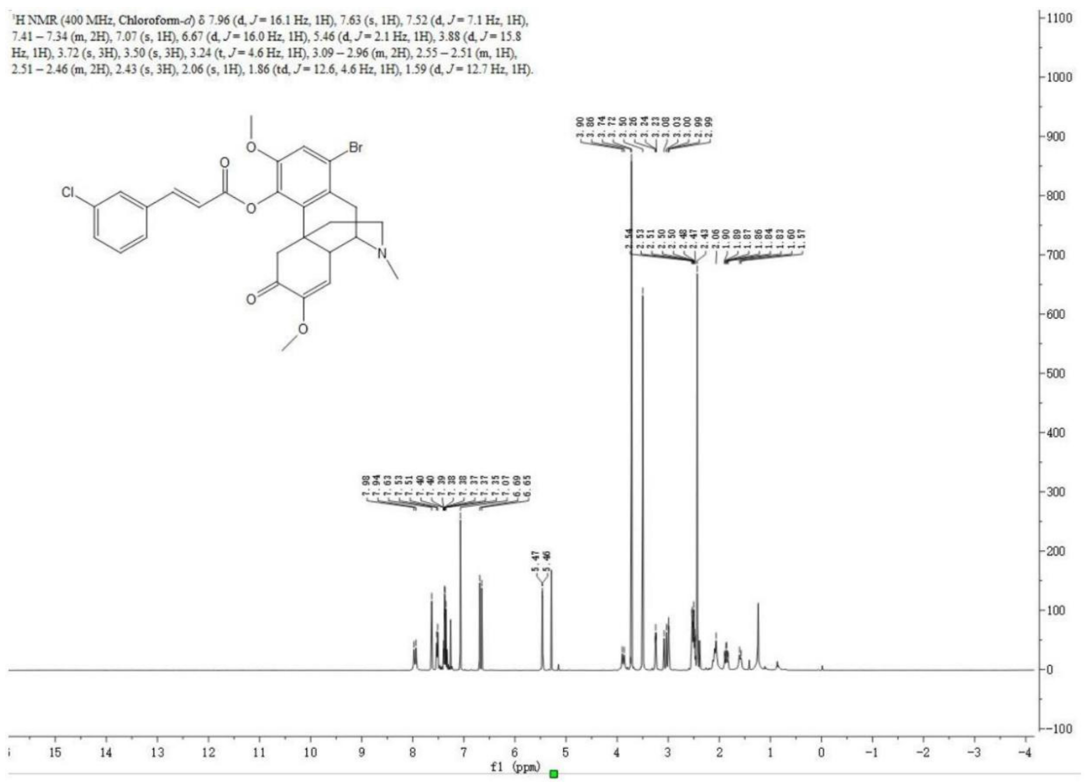

Figure S15. 1H-NMR of 1-Br-4-(3-Cl)-cinnamic acid ester-sinomenine (2d) (deuterated solvent used: $CDCl_3$)

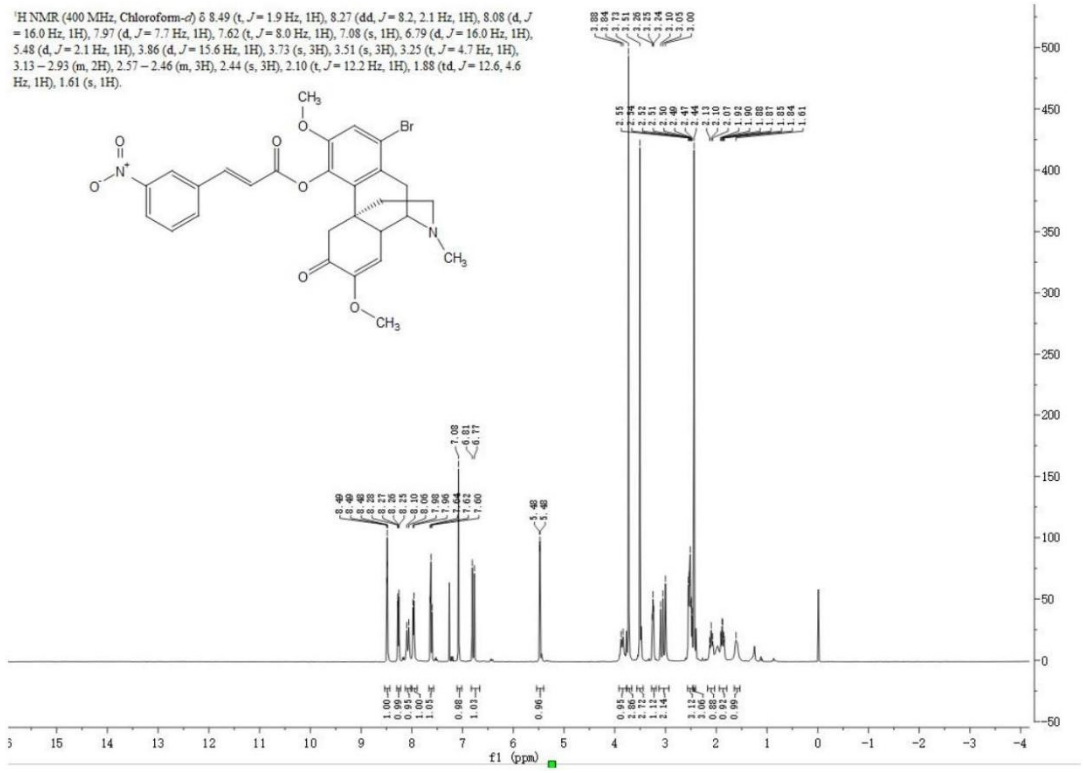

Figure S16. ^{1}H-NMR of 1-Br-4-(3-nitro)-cinnamic acid ester-sinomenine(2e) (deuterated solvent used: $CDCl_3$)

Supplementary Figures ^{13}C-NMR Spectra (compounds 2a–2e, Figures S17–S21)

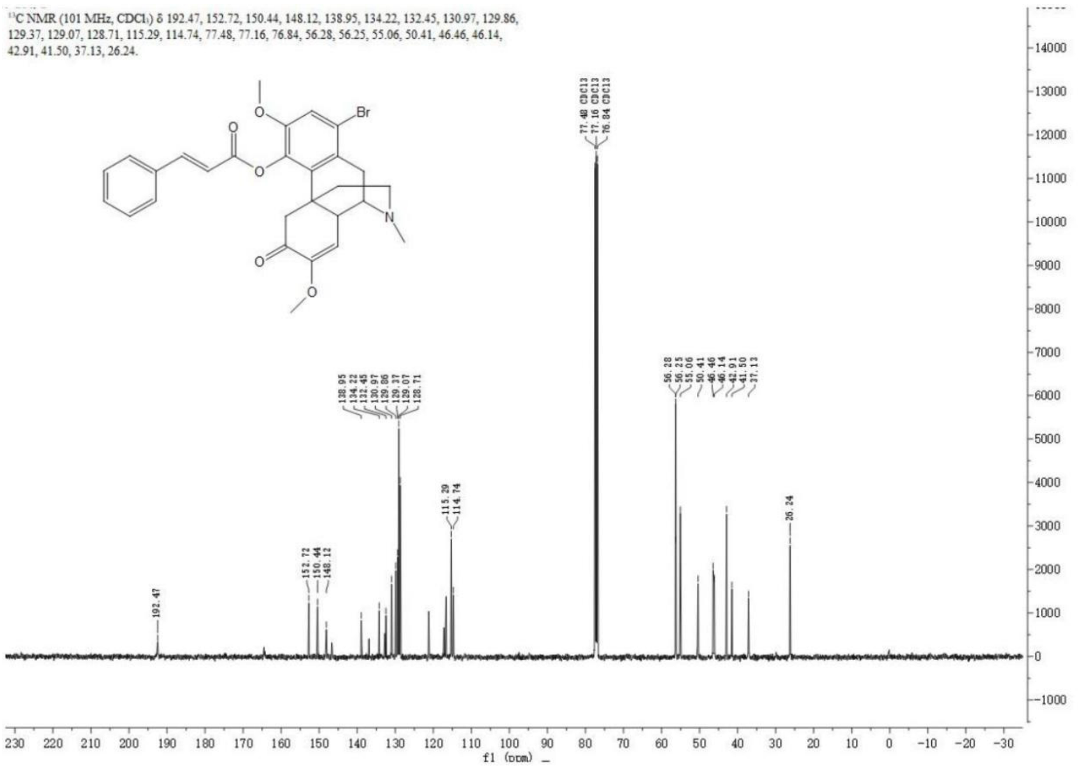

Figure S17. ^{13}C-NMR of 1-Br-4-cinnamic acid ester-sinomenine(2a) (deuterated solvent used: $CDCl_3$)

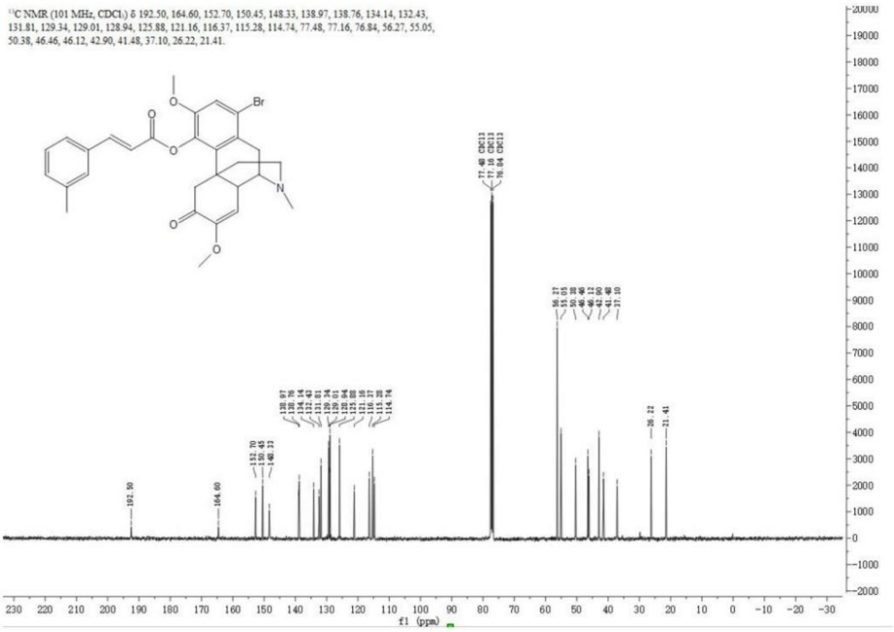

Figure S18. ^{13}C-NMR of 1-Br-4-(3-methyl)-cinnamic acid ester-sinomenine(2b) (deuterated solvent used: $CDCl_3$)

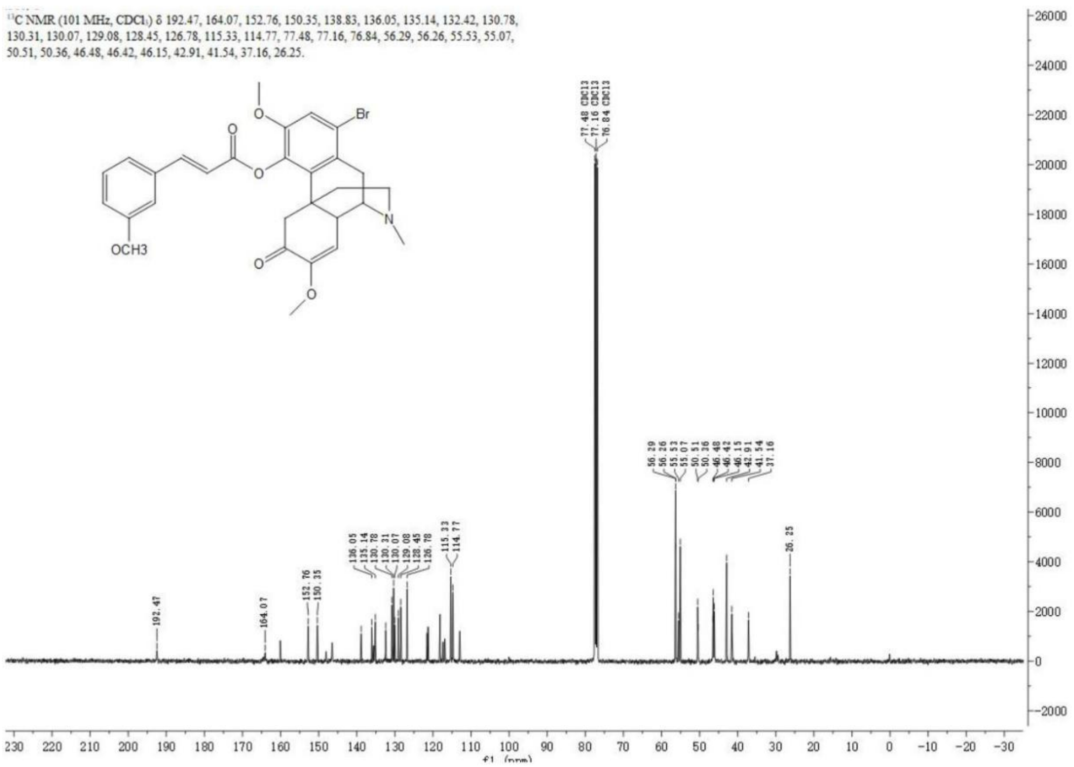

Figure S19. ^{13}C-NMR of 1-Br-4-(3-methoxy)-cinnamic acid ester-sinomenine(2c) (deuterated solvent used: $CDCl_3$)

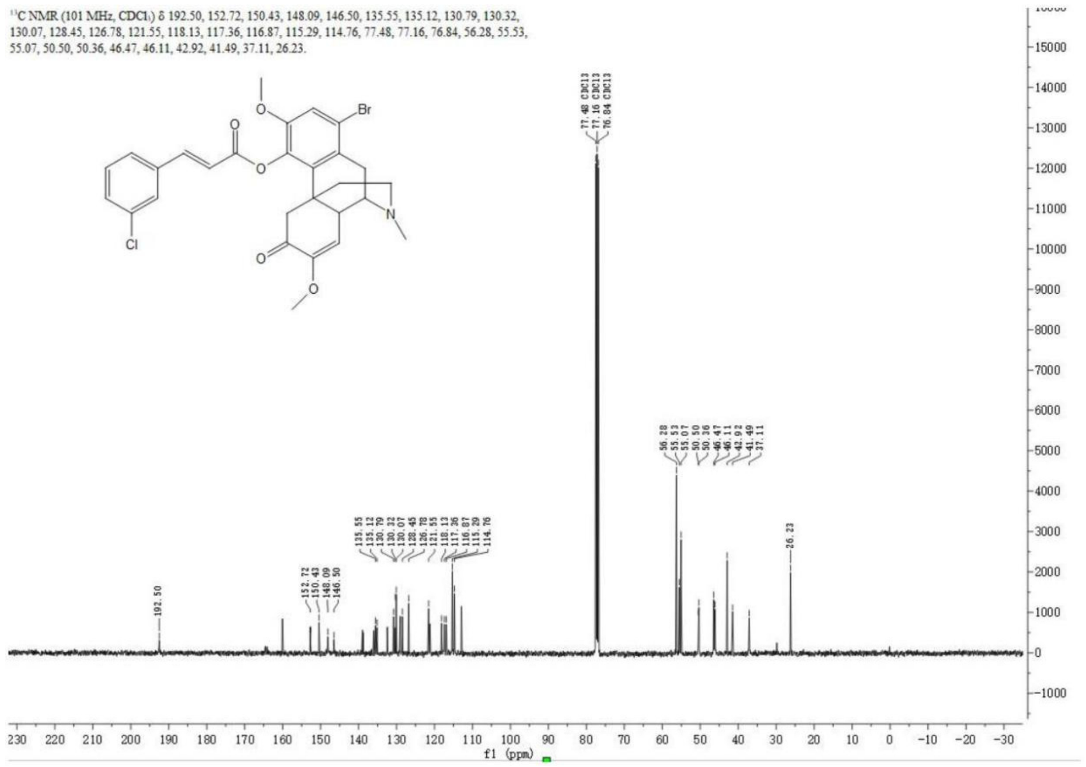

Figure S20. ^{13}C-NMR of 1-Br-4-(3-Cl)-cinnamic acid ester-sinomenine(2d) (deuterated solvent used: $CDCl_3$)

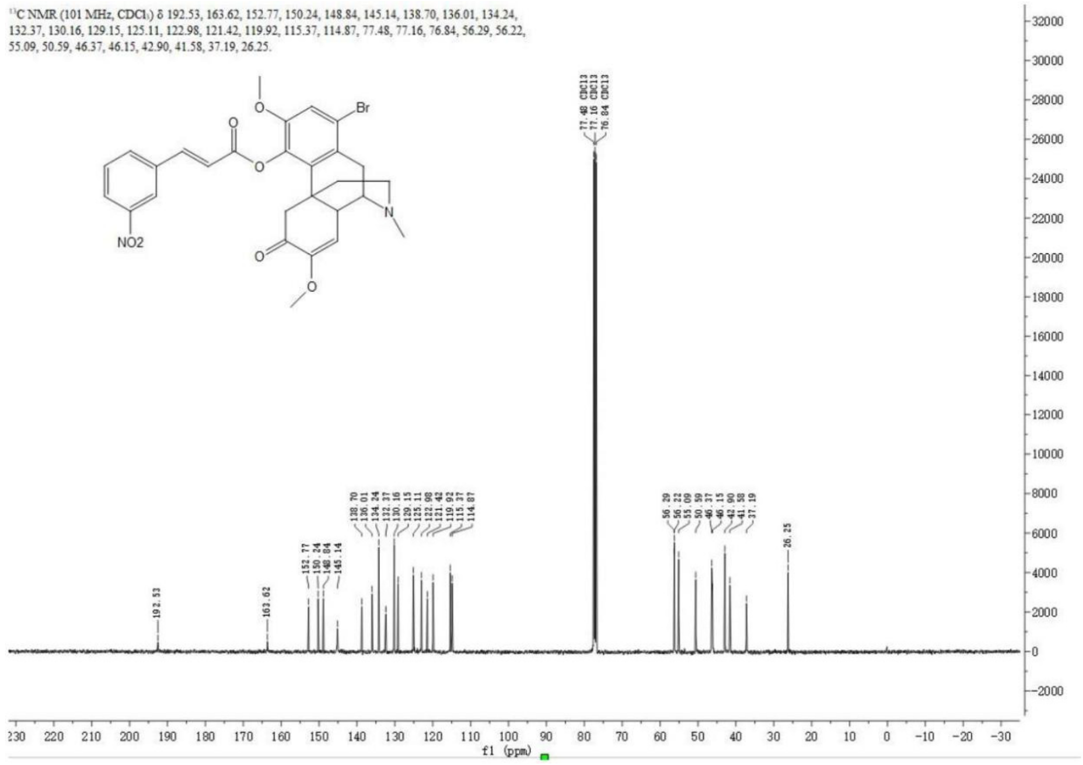

Figure S21. 13C-NMR of 1-Br-4-(3-nitro)-cinnamic acid ester-sinomenine(2e) (deuterated solvent used: $CDCl_3$)

High resolution mass spectrum (HRMS): (compounds 2a–2e, Figures S22–S26)

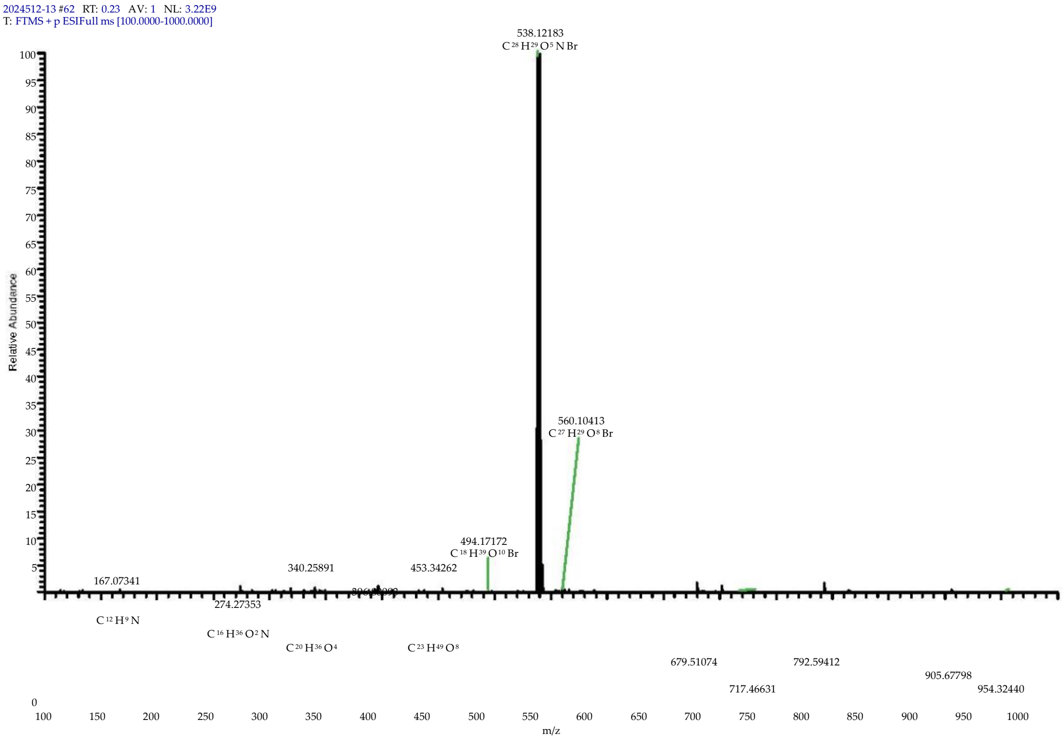

Figure S22. High resolution mass spectrum (HRMS) of compound (2a). The peak at m/z 538.12183

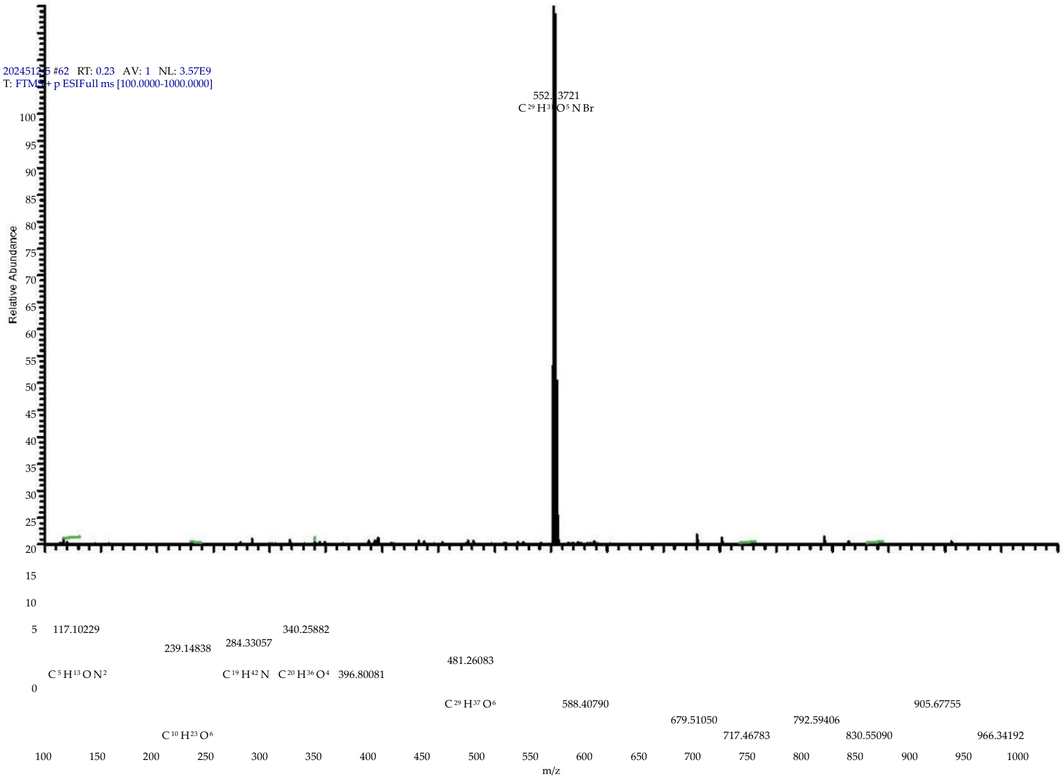

Figure S23. High resolution mass spectrum (HRMS) of compound (2b). The peak at m/z 552.13721

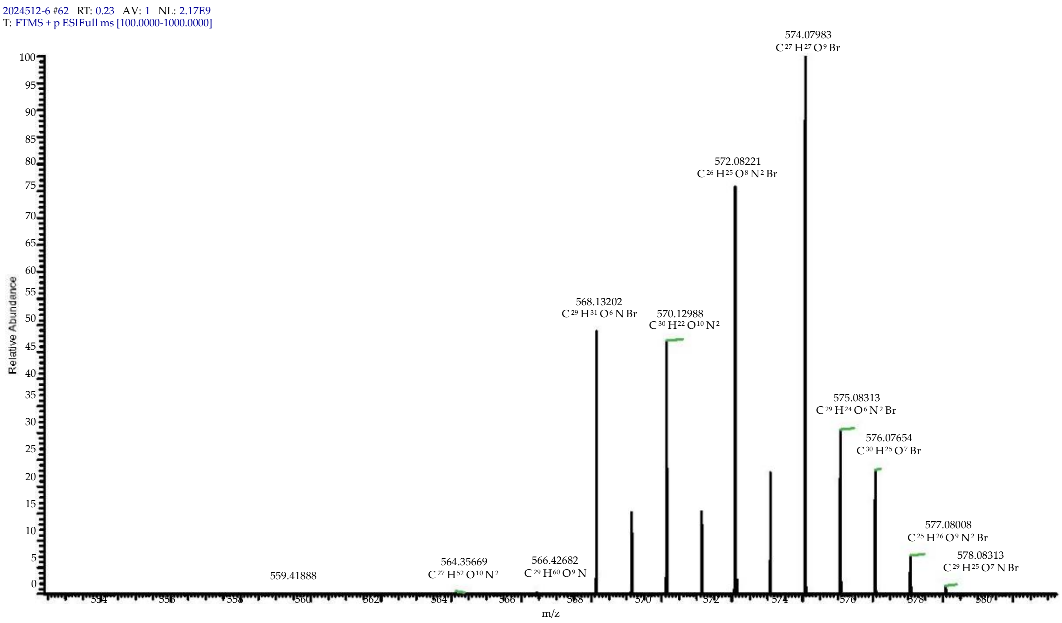

Figure S24. High resolution mass spectrum (HRMS) of compound (2c). The peak at m/z 568.13202

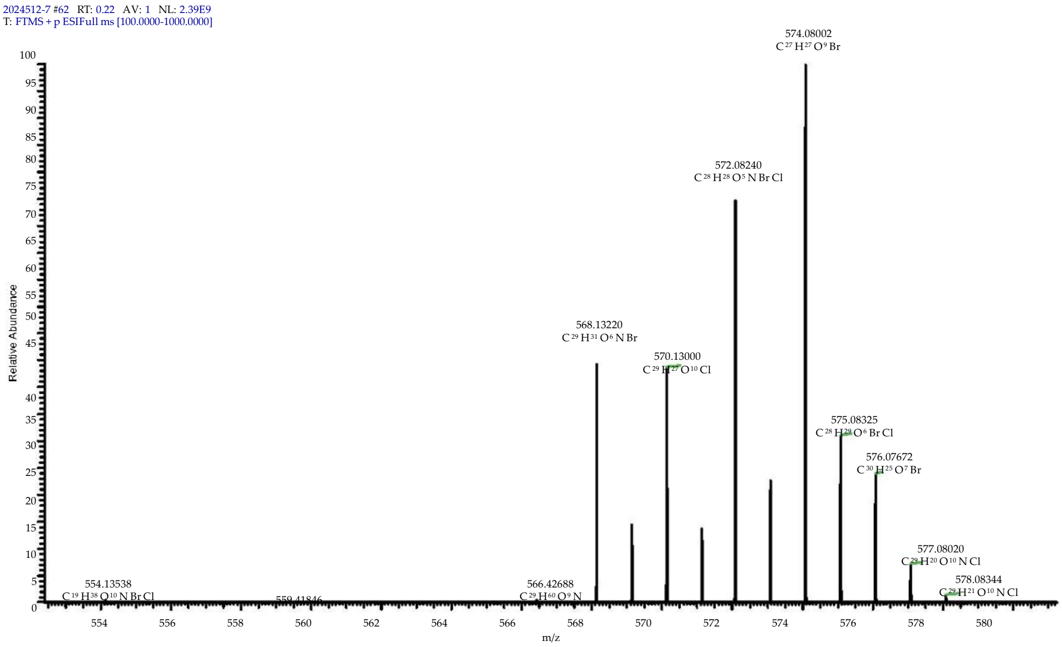

Figure S25. High resolution mass spectrum (HRMS) of compound (2d). The peak at m/z 572.08240

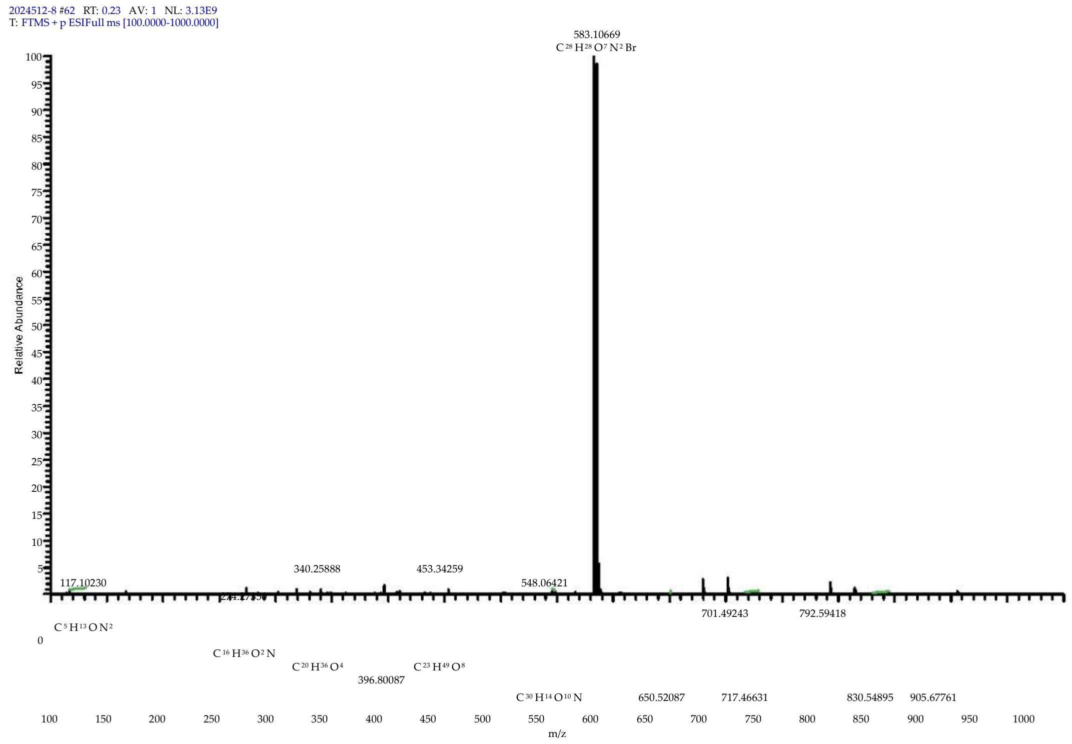

Figure S26. High resolution mass spectrum (HRMS) of compound (2e). The peak at m/z 583.10669
